# Contaminant organisms recorded on plant product imports to South Africa 1994–2019

**DOI:** 10.1038/s41597-021-00869-z

**Published:** 2021-03-16

**Authors:** Davina L. Saccaggi, Melanie Arendse, John R. U. Wilson, John S. Terblanche

**Affiliations:** 1 Plant Health Diagnostic Services, Department of Agriculture, Land Reform and Rural Development, Private Bag X5015, Stellenbosch, 7599 South Africa; 2 Centre for Invasion Biology, Department of Conservation Ecology and Entomology, Faculty of AgriSciences, Stellenbosch University, Private Bag X1, Stellenbosch, 7602 South Africa; 3 Centre for Invasion Biology, Department of Botany and Zoology, Stellenbosch University, Stellenbosch, South Africa; 4 South African National Biodiversity Institute, Kirstenbosch Research Centre, Cape Town, South Africa

**Keywords:** Agroecology, Entomology, Invasive species

## Abstract

Biosecurity interception records are crucial data underlying efforts to predict and manage pest and pathogen introductions. Here we present a dataset containing information on imported plant products inspected by the South African Department of Agriculture’s laboratories between 1994 and 2019 and the contaminant organisms found on them. Samples were received from border inspectors as either propagation material (e.g. plants) or material for immediate use (e.g. fruit). Material for immediate use was further divided into two sample categories, depending on if contaminants were seen/suspected by the border official or not: intervention or audit samples. The final dataset consists of 25,279 records, of which 30% tested positive (i.e. had at least one contaminant) and 13% had multiple contaminants. Of the 13,731 recorded contaminants, fungi (41%), mites (37%) and insects (19%) were most common. This dataset provides insight into the suite of taxa transported along the plant import pathway and provides an important resource for analyses of contaminant organisms in international trade, which can inform strategies for risk assessment, pathway management and biosecurity protocols.

## Abstract (summary)

**Table:** 

Measurement(s)	organism
Technology Type(s)	organism identification assay
Sample Characteristic - Organism	Plantae
Sample Characteristic - Location	Republic of South Africa

Machine-accessible metadata file describing the reported data: 10.6084/m9.figshare.13668824

## Background & Summary

Biological invasions of terrestrial invertebrates and plant pathogens are most often facilitated by global trade of agricultural goods on which these organisms are transported^1,2^. To limit introductions along these pathways, countries apply biosecurity measures to imported goods, including visual inspections at the points of entry^3,4^. Records of inspections and detected organisms provide important information on the taxa transported along these pathways and that are thus at risk of being introduced.

Border interception records have been used historically as a proxy for arrival rates of non-native species and in some cases have been correlated with establishment probability in the new environment^5,6^. Analysis of large interception datasets can improve our understanding of the organisms transported on particular pathways, allowing better risk analyses and biosecurity protocols to be put in place^3,5,7^.

In South Africa, the National Plant Protection Organisation (NPPOZA) within the Department of Agriculture, Land Reform and Rural Development (DALRRD) is responsible for regulating international agricultural trade and enacting biosecurity regulations. Official interception lists are maintained and available on public websites (https://www.dalrrd.gov.za/Branches/Agricultural-Production-Health-Food-Safety/Plant-Health; accessed 15 October 2020). However, only interceptions of listed quarantine organisms, i.e. those that are listed as prohibited organisms on the import conditions for each imported commodity, are recorded. As such, organisms that are already present in South Africa, non-pest organisms (e.g. saprophytes) and organisms of unknown pest status^3^, are not part of the official interception lists. Here we consider both quarantine and non-quarantine organisms found as “contaminants” (sensu Hulme *et al*.^8^, organisms that are unintentionally introduced with a specific commodity). It is important to take note of non-quarantine species, as previously unknown species may become agricultural pests, known species may become pests and other species may become damaging invaders. If future invaders are to be identified and managed, it is therefore important to consider all taxa that are present and moved along these pathways^5^, i.e. the introduction debt^9^. To date, the full record of all organisms detected along the plant import pathway has not been released.

Our goal was to collate, curate, cross-check and share all records related to contaminant organisms found on agricultural plant products (e.g. propagation material, fruit, cuttings and seeds) imported to South Africa from 1994 to 2019.

## Methods

### Sample collection and handling

#### Source of samples to be screened

**Fig. 1:**
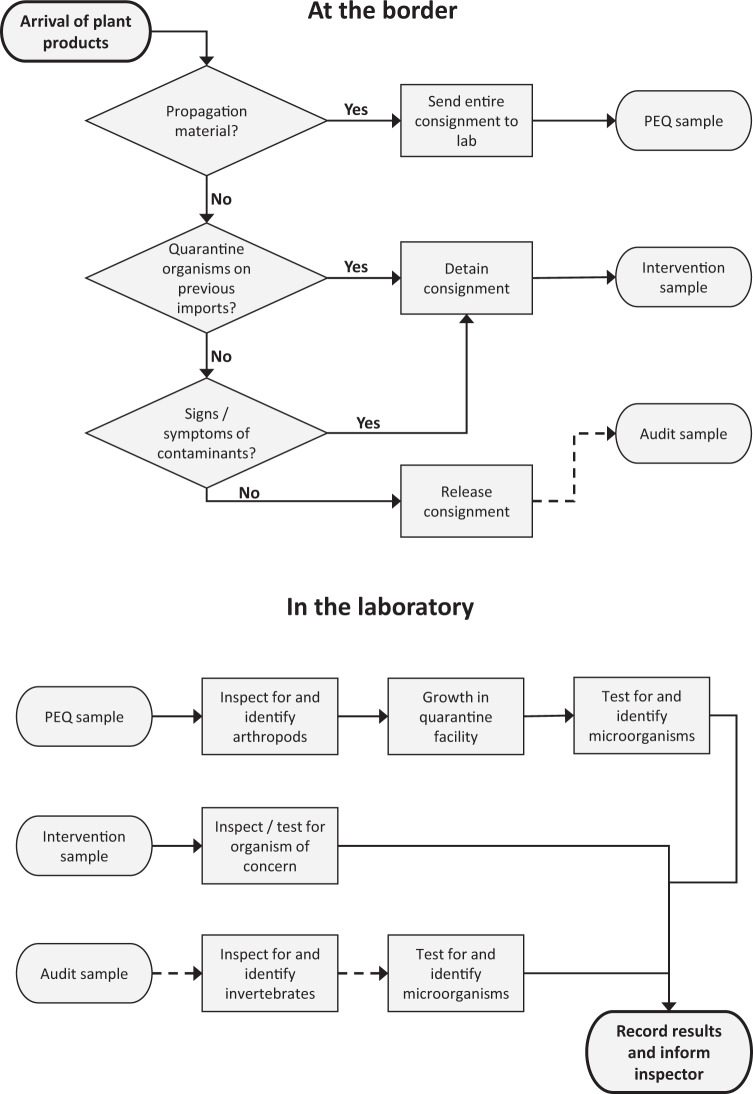
Summary of border and laboratory processes associated with each of the three import sample sources included in this dataset, namely post-entry quarantine (PEQ), intervention and audit samples. Solid lines indicate that these processes are always followed, while dashed lines indicate that the process is sometimes followed. PEQ samples are received from plant propagation or nursery material that needs to be quarantined upon arrival. Intervention samples are received from consignments in which the border inspector finds or suspects a pest or pathogen. Audit samples are ad hoc samples drawn from consignments that show no sign of contamination. These sample sources are explained in more detail in the text.

South Africa currently has 72 official points of entry—8 seaports, 10 airports and 54 land border posts^10^. The DALRRD has border inspectors at most of these points (although staffing levels have varied considerably). DALRRD border inspectors inspect goods and travellers entering the country for plant contaminants. As part of DALRRD’s biosecurity protocol, three types of samples are collected and sent to DALRRD laboratories in Stellenbosch or Pretoria for further investigation (Fig. 1).

1. Intervention samples. If the border inspector finds or suspects a pest or pathogen in a consignment, he/she will take a sample and send it to one of DALRRD’s diagnostic laboratories. A suspicion of contamination is often the result of quarantine organisms being detected on previous consignments of the same commodity. The imported consignment is detained at the border until laboratory results are completed. Due to the time-sensitivity of such imports, the samples are usually only inspected or tested for the taxa of concern.
2. Audit samples. As above, these samples are drawn from consignments of plant products for immediate use. However, they are drawn on an ad hoc (haphazard) basis from consignments that show no signs of contamination during border inspections. In the laboratory, these samples are often inspected or tested for multiple taxa.
3. Post-entry quarantine (PEQ) samples. Plant products for propagation purposes or nursery material (e.g. *in vitro* plantlets, seedlings, budwood) are shipped in sealed packages and transported directly to DALRRD’s agricultural quarantine facilities. For small consignments (under 50 units), all units in the consignment are tested and inspected by laboratory officials. For larger consignments, random samples are drawn and inspected following a hypergeometric sampling protocol^11^. Inspection for arthropods and initial examination for micro-organisms takes place in a biosecurity containment facility (see Saccaggi & Pieterse^12^ for further details). The material is then grown in a dedicated quarantine facility and further testing for pathogens takes place when the plants are in active growth.

### Taxa inspection, testing and identification methods

All inspections, testing and identifications are carried out by DALRRD laboratory officials specialised in each taxonomic group. Taxonomic identifications are routinely done by DALRRD officials, taxonomists at the Biosystematics Division of the South African Agricultural Research Council (ARC) or higher education institutions, depending on the expertise available at the time. All recorded identifications in the dataset were retained, regardless of level of identification or biosecurity status of the organism. It should, however, be noted that all organisms found were not always recorded (see below for further explanation).

Arthropods (mostly insects and mites) and Molluscs are detected via visual inspection using a stereo-microscope. For these taxa, all organisms detected are recorded. Organisms are most commonly identified morphologically, with molecular identification being performed for certain groups. Identification is performed to the point at which a reasonable phytosanitary decision can be made (i.e. sometimes taxonomic precision is sacrificed for time and/or resource efficiency and logistic reasons). Thus specimens from predatory or saprophytic groups are often only identified to family or genus, while specimens within plant-feeding groups are identified to species where possible.

Nematodes are detected by extraction from samples using relevant extraction methods. Saprophytic and predatory nematodes are sometimes noted, but often ignored as they are not considered to be of phytosanitary concern. Plant-feeding nematodes are identified morphologically to species where possible.

Fungi and Bacteria are detected visually in the growing plant, as well as by conventional isolation and plating techniques, followed by biochemical tests and/or morphological identification. Some targeted pathogens are detected and identified by molecular techniques such as PCR and DNA sequencing. Saprophytic or secondary fungi or bacteria are sometimes noted, but often not recorded as part of the sample record.

Viruses are screened for by immunological techniques, notably ELISA and hardwood and herbaceous indexing. ELISA techniques detect a target virus of concern and give no information as to the presence or absence of other viruses in the sample. Hardwood and herbaceous indexing are used to determine if any graft- or mechanically-transmissible viruses are present in the sample, although these methods cannot be used to determine the viruses’ identity.

Phytoplasma screening is done by nested PCR designed to detect any phytoplasma. On specific crops, phytoplasma groups are detected by using targeted PCR methods. If necessary, sequencing of PCR products is used for more specific identification.

### Data collection and handling

Metadata for samples were recorded by the border inspector before submission to DALRRD’s laboratories. Ideally, he/she recorded geographic origin of the commodity, crop and sample type, date of collection, details of importer and exporter, organisms to test for and any additional observations. However, in practice, this information was not always recorded in full. See Tables 1, 2 and 3 for more details on information included in the dataset. Due to the sensitivity of this kind of trade data, some of the data in the current dataset are grouped or anonymised to protect confidentiality. In particular, import date is only listed as month and year and the names of importers and exporters are removed.

**Table 1:** A summary of information fields and descriptions for each imported sample recorded in the South African plant import dataset used in the datasheet “List of contaminants on SA plant imports 1994–2019.csv”^23^.

Field name	Field description	Standardisation reference	Editing notes
catalogNumber	A unique numerical identifier	NA	This has been added from the original laboratory number in order to anonymize the data. If needed, DS can conduct a trace-back to the original DALRRD laboratory number.
year	Year in which sample was received	NA	Dates were checked chronologically by sample number and incorrect dates corrected (e.g. day & month swapped) Due to confidentiality concerns, DALRRD has decided not to publish exact dates.
month	Month in which sample was received		
sample.source	Source of sample	Three sample categories: 1) intervention; 2) audit; 3) post-entry quarantine (PEQ). (see text and Fig. 1 for more detail)	
country	Country from which the sample originated	United Nations list of accepted country names: https://unstats.un.org/unsd/methodology/m49	The recording of a sub-country location does not necessarily mean that the sample originated from that location. Therefore when city or state was listed, it was replaced with country.
higherGeography	Geographic region	United Nations M49 standard geoscheme: https://unstats.un.org/unsd/methodology/m49	
crop.vernacularName	Crop, as listed by the border inspector		Either the vernacular or scientific name was added, as needed.
crop.scientificName			
crop.family	Taxonomic grouping	GBIF (search: 28 Aug 2020)	Where multiple crops were listed, order and/or family was included if these were the same, but recorded as “multiple” if they were not.
crop.order			
crop.commodity	Commodity type, as recorded by the border inspector	Table 3: List of import commodity types	The original 9 categories were expanded to 30 and assigned to category based on what was recorded as commodity and crop as well as any additional laboratory records or expert experience.
importer.code	A unique alphanumerical identifier for importer	NA	Anonymization of these data are necessary to protect the confidentiality of the trading companies.
exporter.code			
Contaminants (79 columns)	Contaminants, arranged by order or suborder (where appropriate), plus extra columns for unidentified higher taxa	GBIF (search: 29 July 2020) Recorded in Table 2.	Where taxonomy could not be resolved using GBIF, the taxonomy originally recorded was retained.

More details are provided in Supplementary Table 1.

**Table 2:** Information fields and descriptions for taxa information associated with contaminant organisms detected on import samples received by South Africa used in the datasheet “Metadata of contaminants on SA plant imports 1994–2019.csv”^23^.

Field name	Field description	Standardisation reference	Standardisation notes
Taxon	The group into which the taxa fall: insects, mites, other arthropods, molluscs, nematodes, fungi, bacteria and viruses.		
phylum	Classification. Where it was unknown, it is recorded as “undet”.	Classification according to the GBIF taxonomic backbone (search: 29 July 2020)	Where taxonomy could not be resolved using GBIF, the taxonomy originally recorded was retained.
class			
order			
family			
genus			
scientificName			
number.of.records	Number of records of each taxon in the current dataset		
SA.occurenceStatus.historical	Presence or absence of species in South Africa at the time of recording.	see column ‘SA.occurenceSources’	
SA.occurenceStatus.current	Presence or absence of species in South Africa in 2020 and year of first recording, if absent at time of recording.		
SA.occurenceRemarks	Additional remarks concerning occurrence.		Occasionally published sources are incorrect and this is noted.
SA.occurenceSources	Sources consulted for occurrence status	GBIF^13^; CABI^18–20^; Catalogue of Life^21^; SANBI animal species checklists^22^; and other sources^29–36^.	Where no records of occurrence in South Africa could be found, the species was listed as absent.
SA.regulatoryStatus	Whether the species is included on South Africa’s quarantine lists.		
taxonRemarks	Additional remarks regarding taxonomy or classification.		When sources have given occurrence data for the species under a different name, that name is mentioned.

**Table 3:** List of import commodity types used in the datasheet “List of contamiants on SA plant imports 1994–2019.csv”^23^. The original categories listed by the inspectors were expanded to 30 commodity types based on additional laboratory information and expert experience.

Original dataset category	Commodity type category
Fruit	Dried fruit
	Fresh fruit
Seeds	Beans
	Grain
	Nuts
	Seeds
Soil	Soil
	Growing medium
Bulbs	Bulbs
Roots	Rhizomes
	Roots
	Tubers
Cuttings	Canes
	Cut Flowers
	Cuttings: budwood
	Cuttings: leafy
	Cuttings: rooted
	Cuttings: rooted budwood
Plants	*Ex vitro* plantlets
	*In vitro* plantlets
	Plantlets
	Plants
Foliage	Fresh foliage
	Dried foliage
Other	Animal feed
	Dried spice
	Manufactured goods
	Pollen
	Wood
	Unknown

Electronic databases of samples received by the DALRRD laboratories were maintained by the laboratory staff. These databases were not official departmental databases and therefore did not need to include information relevant to other sections involved in biosecurity. For instance, total number of imports, total size of each consignment, observations of the inspector, details of phytosanitary certificates and release or detention of the consignment were never recorded. The databases also included samples processed by the laboratory for export or for national pest surveys. Partly due to their unofficial status, the databases were transient, with new databases started once software became outdated, the old one became too big or when new categories or information were to be included. For this study, we collated, curated and cross-checked information from nine of these databases, spanning 26 years from 1994 to 2019.

Recorded laboratory data varied between taxa and over time and as priorities and understanding of biosecurity changed. In the initial years considered here (ca. 1994–2000), the focus was on pests or pathogens of quarantine importance, i.e. those on the prohibited list. Other organisms found on samples were not consistently recorded and, when they were, they were often recorded in broad groupings (e.g. “saprophytic nematodes”). More recently, there has been a shift towards recording all organisms detected, but this has still not been done consistently [although from ~2005 onwards the officials responsible for arthropods and molluscs have tried to record everything found (DS, MA personal observations)]. Thus prohibited (i.e. quarantine organisms) were always recorded, but the recording of other contaminants was inconsistent.

Data clean-up started with collation of all data from the nine databases. Initially, these contained 99,023 records, with 50,655 recorded as imports, 31,163 as exports, 11,004 as surveys with the remaining 6,201 falling into other categories or uncategorised. Only imports were retained, as this was the only category of interest for this study. For some imports, sample information was recorded in one database, while results of inspections/tests for different taxa were recorded in other databases. Thus a single sample could have up to four duplicate records. Each of these was checked individually and collated into one record for the sample. Spelling mistakes, incorrectly recorded information (e.g. information recorded in the wrong field) and missing information were traced back through paper records and corrected wherever possible. If the original data could not be found, these ambiguous records were excluded. After this data clean-up, the dataset comprised a list of 26,291 import records, of which 2,572 resulted from intervention samples (sample source 1 above, Fig. 1), 10,629 were audit samples (sample source 2 above, Fig. 1) and 13,090 were PEQ samples (sample source 3 above, Fig. 1). Data clean-up then continued for the organisms found on the imported samples.

Taxon names were extracted and spelling and classification were corrected and/or added by hand. The list of taxa was checked against the Global Biodiversity Information Facility (GBIF)^13^ using the software package ‘rgbif’^14^ in Rstudio version 1.3.959^15^ running R version 4.0.2^16^. This highlighted additional spelling mistakes and provided a taxonomic backbone to work from. The classification of a number of taxa had changed over the years and thus using a common taxonomic backbone was needed for consistency. Some taxa, most notably some mite species, could not be found on GBIF. In these cases, the taxonomy provided by the taxonomist who initially identified the organism was retained. Virus taxonomic information was also not available on GBIF and the database of the International Committee on Taxonomy of Viruses (ICTV) was used^17^.

Species occurrence in South Africa was determined by consulting published species distribution lists. The following data sources were consulted: GBIF^13^ (accessed 29 July and 03 Aug 2020); CABI Crop Pest Compendiums and Invasive Species Compendium^18–20^; the Catalogue of Life^21^; animal species checklists published by the South African Biodiversity Institute (SANBI)^22^; and for any remaining species internet searches were conducted for literature citing distributions (listed in Table 2).

In South Africa, lists of organisms prohibited from entering the country have been compiled by DALRRD and the Department of Forestry, Fisheries and the Environment (DFFtE). DFFtE’s list of prohibited species focussed mostly on organisms of environmental concern, although some prohibited organisms were also of agricultural concern, while DALRRD is only concerned with agricultural pests. DALRRD issues import permits for each unique crop, commodity and country combination from which plant products originate. Thus there is no single consolidated quarantine list for South Africa. Furthermore, any quarantine list is not static, but needs to change as species’ distributions, taxonomic revisions or pest status changes. Thus it is very difficult to provide a list of which detected organisms are of quarantine status to South Africa at any given time and particularly in a dataset spanning 26 years. As far as possible, we have indicated the regulatory status of the species in the datasheet “Metadata of contaminants on SA plant imports 1994–2019.csv”^23^. This regulatory status would have been of critical importance to inform contemporary phytosanitary decisions. However, given that such lists are dynamic and a core aim of presenting these data is to facilitate analyses of future invaders^9^, it is important to present information on all organisms detected. Moreover, this allows a more comprehensive assessment of the role of different pathways and will facilitate comparisons with other countries.

## Data Records

### Structure of dataset

Two datasheets are archived and available from figshare.com as semicolon-delimited files (.csv)^23^.

The first datasheet, “List of contaminants on SA plant imports 1994–2019.csv”^23^, comprises laboratory records of samples of imports received by South Africa between 1994 and 2019 and the contaminant organisms recorded on them, as recorded by DALRRD diagnostic laboratories. Each line represents records for a single sample. The first 13 fields are information relating to the sample and are described in Table 1. The following 79 fields contain information relating to any organisms recorded on the sample, arranged according to order or, where appropriate, suborder or superfamily (Table 1). An NA in these columns means that the sample was not inspected or tested for that particular organism. A zero (0) means that the sample was inspected/tested, but no contaminant organism (of that classification) was found.

The second datasheet, “Metadata of contaminants on SA plant imports 1994–2019.csv”^23^, comprises more details related to the contaminant organisms listed in the first datasheet, including taxonomy and number of records in the dataset. Information is also included as to whether the species listed has been recorded in South Africa at the time of the record, if it has since established and any other additional notes. The information contained in this datasheet is summarised in Table 2.

Parts of this dataset have been used previously to discuss specific plant-feeding insects and mites detected on imports^12,24,25^ and for discussion of introduction pathways when reporting introductions of new species to South Africa^26^.

### Summary of dataset

The figures and tables shown here present a summary of the information available in the dataset and some examples of what these data could be used for. Between 1994 and 2019, 26,291 import samples were recorded by DALRRD laboratories.

**Fig. 2:**
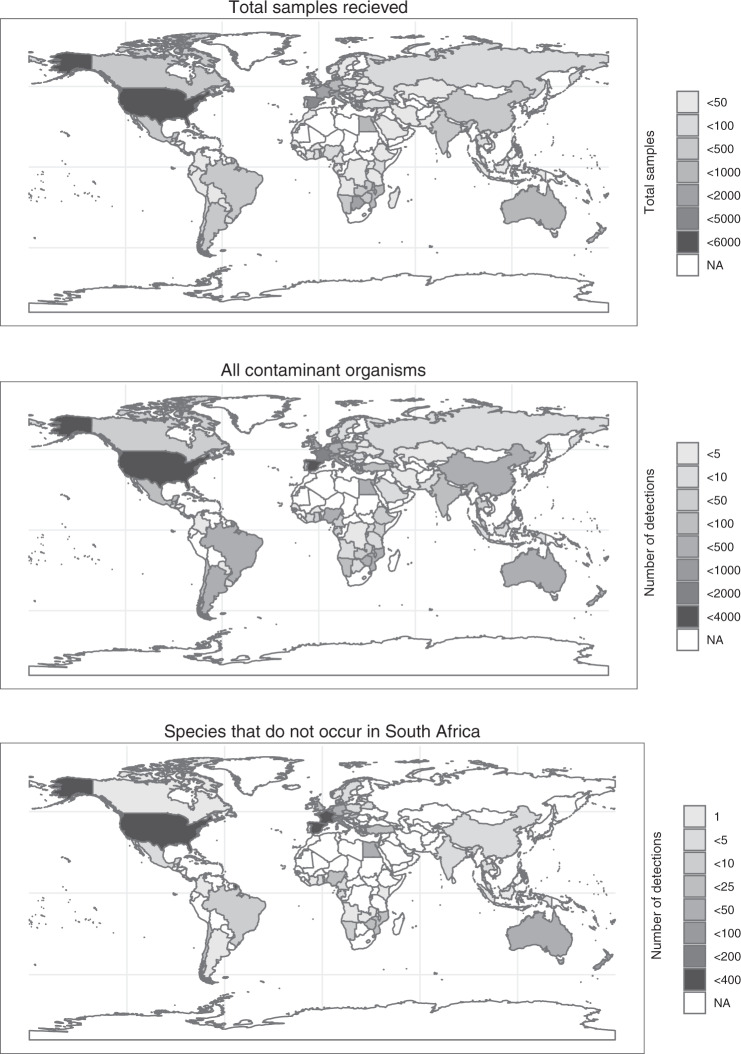
Import samples examined by the South African diagnostic laboratories between 1994 and 2019 from different countries. Top: All samples received. Middle: Contaminant organisms recorded on these samples. Bottom: Detections of species that were not present in South Africa at the time of recording. NA (white colour) indicates that no samples (top) or no contaminant organisms (middle and bottom) were received from these countries.

Import samples originated from 95 recognized countries (as per the UN geoscheme) and an additional 15 major subregions (e.g. Hawaii, Sicily) in all world regions (Fig. 2). The proportion of positive samples and the number of contaminant organisms detected varied between regions, with a predominance of samples from Europe and the USA (Fig. 2).

**Fig. 3:**
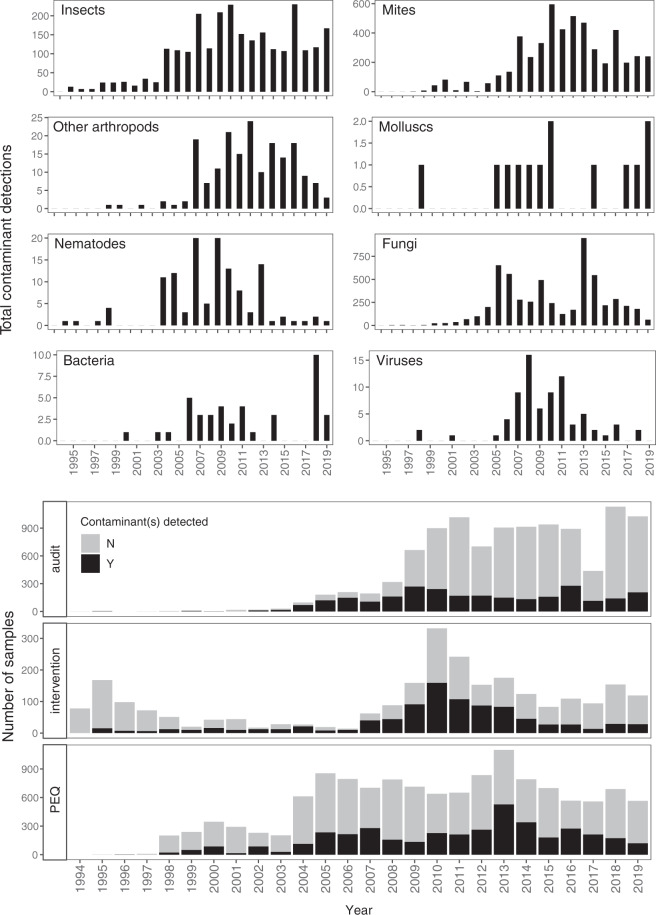
Trends in the number of plant product samples received and contaminant taxa detected by the South African DALRRD diagnostic laboratories between 1994 and 2019. The top eight panels show the total numbers of each taxonomic group that were intercepted. The bottom three panels show the sample types received annually (see text and Fig. 1 for more explanation) and the proportion of these on which contaminant organisms were detected. Note the different scales on the vertical axes.

Of the samples inspected/tested, 29% (7,520) were host to at least one contaminant organism (i.e. a positive sample) (Fig. 3). Numbers of samples received, number of positive samples and proportions of different taxa detected varied over time. 13,731 detections of contaminant organisms were recorded, with multiple contaminants often present on the same sample (Fig. 3).

Organisms in 13 phyla and 27 classes were recorded (Table 4). Fungi (including Chromista) (42%), Acari (mites and ticks) (37%) and insects (19%) predominated. Overall, only 28.4% of detected organisms were identified to species level (Table 4). However, the level to which detected organisms were identified varied as explained previously. For instance, 99.1% of fungi were identified at least to genus level but only 4.7% to species level; whereas 49.3% of insects and 43.4% of mites were identified to species level (Table 4). Of the species recorded, 46% were absent from South Africa at the time they were detected (Table 4). Seven of these species have since established in South Africa (datasheet 2: “Metadata of contaminants on SA plant imports 1994–2019.csv”^23^). Only 53 of the 225 species recorded as absent from South Africa (Table 4) were listed as prohibited organisms at the time of their detection (datasheet 2: “Metadata of contaminants on SA plant imports 1994–2019.csv”^23^).

**Table 4:** A summary of the number of taxa, species not present in South Africa and the number of individual records relating to each taxon/category in this dataset^23^.

Higher taxon	Number of taxa in dataset (number of records identified to at least this classification level):						
	Phyla	Classes	Orders	Families	Genera	Species	Species not in SA
Bacteria (n = 41)	3 (41)	4 (41)	9 (41)	10 (41)	14 (41)	11 (17)	3 (6)
Fungi (n = 5,658)	3 (5,651)	8 (5,640)	20 (5,640)	34 (5,640)	56 (5,640)	30 (267)	10 (48)
Chromista (n = 34)	1 (34)	1 (34)	1 (34)	2 (34)	2 (34)	4 (5)	0 (0)
Viruses (n = 76)	3 (72)	4 (72)	5 (72)	6 (72)	6 (72)	13 (72)	5 (37)
Invertebrata (n = 7,922)	Arthropoda (7,785)	Insecta (2,547)	11 (2,530)	107 (2,286)	221 (1,429)	234 (1,255)	82 (210)
		Acari (5,054)	5 (4,879)	35 (4,417)	108 (3,266)	151 (2,193)	107 (1,227)
		Arachnida (54)	2 (54)	9 (40)	15 (32)	5 (7)	2 (2)
		Chilopoda (4)	0 (0)	0 (0)	0 (0)	0 (0)	0 (0)
		Entognatha (125)	1 (125)	1 (2)	0 (0)	0 (0)	0 (0)
		Malacostraca (1)	1 (1)	1 (1)	0 (0)	0 (0)	0 (0)
	Mollusca (13)	2 (13)	3 (12)	7 (12)	8 (12)	7 (10)	2 (2)
	Nematoda (124)	2 (118)	5 (118)	16 (118)	17 (111)	34 (67)	14 (28)
**TOTAL**	**13 (13,720)**	**27 (13,703)**	**63 (13,506)**	**228 (12,663)**	**447 (10,637)**	**489 (3,893)**	**225 (1,560)**

Note that species not in South Africa refers to the status at the time of detection in the dataset. Seven of these species have since established in the country^23^.

### Limitations of dataset

The dataset provided here is a valuable source of information about organisms transported on internationally traded agricultural plant products. However, the dataset has some notable limitations.

Firstly, only samples received by the DALRRD laboratories are included. The exact methods of drawing these samples from imported consignments (be they intervention, audit or PEQ samples) were never recorded, but will have varied over time as priorities and capacity varied. Thus this dataset is an under-estimate of the rates of interception and likely a gross under-estimate of rates of introduction of species to South Africa along the contaminant pathway.

Secondly, methods of recording varied between taxa and over time. For instance, before ca. 2005 only organisms of quarantine importance were recorded for most taxa, with other organisms detected either being recorded as a group (e.g. “saprophytic nematodes”) or not at all (e.g. “No fungi of phytosanitary importance were found”). In the present dataset, these finding are recorded as zeros (0) – but it may be that other non-phytosanitary organisms were found but not recorded.

Thirdly, methods of detection and identification are of necessity different for different taxonomic groups and the full suite of tests performed was not always recorded. For instance, viruses were detected by PCR or ELISA protocols developed to test for specific species. Results from these tests indicate which of these species were present, but give no information as to whether other (untested) species may also have been present. By contrast, arthropods were detected using a stereomicroscope, but who was doing the inspection and how thoroughly they did it will vary and there will likely be a bias towards conspicuous or abundant contaminants.

Fourth, the presence of a taxon in the dataset does not imply that that taxon is present in South Africa, nor can inclusion in this dataset be considered a report of occurrence status in another country. These data are simply interceptions and on their own are insufficient for phytosanitary decision-making. Similarly, the absence of a taxon from this dataset does not imply that it has not been imported to South Africa over this time period or that it has not reached South Africa. South Africa has several extensive land borders and it is believed that several pests and diseases have spread from South Africa to the rest of Africa and vice versa^27^. A detailed assessment of the role of contaminants in the introduction of alien species to the region would require combining biosecurity information from all the countries in the region^28^.

## Technical Validation

### Record verification

Records were collated, curated, cross-checked, verified and corrected from nine electronic laboratory datasets. Record verification was performed by DS, who has worked in the DALRRD diagnostic laboratories since 2006 and therefore is intimately familiar with the sampling and recording procedures employed. Wherever necessary, she enlisted the help of more senior DALRRD employees to clarify records, sampling procedures or taxonomy. If the information recorded in the databases was ambiguous or did not match between records in different databases, the sample was traced back using the original paper records as far as possible. Samples which could not be verified in this way were excluded from publication in the current dataset. The catalog number supplied with this dataset can be traced back to the original record in the DALRRD databases if needed.

### Accuracy and trace-ability of identifications

All taxa were identified by highly experienced specialists in each taxonomic field and were as accurate as possible at the time. However, it must be noted that changes in taxonomy and experience could influence the accuracy of identification. See Craemer & Saccaggi^25^ for an example of this. When a potential quarantine pest was identified, the identification was always verified by at least two independent experts, usually by DALRRD and the ARC. The databases collated here did not include the name of the person who performed the identification, but this information is usually available on paper records should a trace-back be needed. Similarly, many (but not all) specimens were retained in reference collections at DALRRD or incorporated into the National Collections of the Biosystematics Division of the ARC (https://www.arc.agric.za/arc-ppri/Pages/Biosystematics.aspx).

## Usage Notes

The dataset presented here details contaminant organisms found on internationally traded goods and was the result of non-random and variable sampling and recording methods. These caveats apply to most historical interception, contaminant or pathway datasets and we therefore encourage authors wishing to use such a dataset to consult a person intimately familiar with the background of that particular dataset. For this South African dataset, DS can be contacted.

Some elements of the dataset have been anonymised to protect confidentiality, most notably names of importers and exporters and exact date of entry of an imported consignment. The catalog number provided with the current dataset can be used to trace back to the original record in the DALRRD databases. Should there be any queries in this regard, DS or MA can be contacted.

## Supplementary information

Supplementary Table 1
