# Supplementary material for: Contaminant organisms recorded on plant product imports to South Africa 1994–2019
Source: Sci Data. 2021 Mar 16;8:83. doi: 10.1038/s41597-021-00869-z (PMC7966792; doi:10.1038/s41597-021-00869-z)
Supplement: Supplementary file 1 — Supplementary Table 1 [file 41597_2021_869_MOESM1_ESM.docx]

**Supplementary Table 1:** Information fields and descriptions for each imported sample recorded in the South African plant import dataset.

| **Field name** | **Field description** | **Original format** | **Standardisation reference** | **Standardisation notes** | **Other editing notes** |
| --- | --- | --- | --- | --- | --- |
| **catalogNumber** | A unique numerical identifier | NA | NA | NA | This has been added from the original laboratory number in order to anonymize the data. If needed, DS can conduct a trace-back to the original DALRRD laboratory number. |
| **year** | Year in which sample was received | Date on which sample was received by the laboratory. In a number of instances, dates were incorrect due to formatting errors (e.g. day/month swapped; year entered as 2-digits and then mistaken for day/month). | NA | Dates were checked chronologically by sample number, and incorrect dates corrected (e.g. day & month swapped) | Due to confidentiality concerns, DALRRD has decided not to publish exact dates. |
| **month** | Month in which sample was received |  |  |  |  |
| **sample.source** | Source of sample | In earlier datasets, samples were listed as "import: payable" or "not-payable", which was equivalent to "import ID" and "import audit" in later datasets. In later datasets, the border inspector choose sample type from a number of categories, including "import ID", "import audit", "quarantine ID" and "quarantine audit". | Three categories of imports were identified: 1) intervention samples; 2) audit samples; 3) post-entry quarantine (PEQ) samples. (see text and Figure 1 for more detail) | NA | NA |
| **country** | Country from which the sample originated | Usually country, but sometimes city or state was listed. Entries were in English or Afrikaans, sometimes abbreviated, sometimes misspelt. | United Nations list of accepted country names:https://unstats.un.org/unsd/methodology/m49 | The recording of a sub-country location does not necessarily mean that the sample originated from that location, but could mean that the final packaging was done there, or that the headquarters of the exporter were located there (i.e. the address listed on the phytosanitary certificate). To avoid this ambiguity, when city or state was listed it was replaced with country. | A few imports were recorded as originating from Yugoslavia, despite its dissolution in 1992 (i.e. before the first sample recorded in the current dataset).In other cases where a country has changed name, all records were updated to the current name (e.g. Swaziland to Eswatini). |
| **higherGeography** | Geographic region | This was not part of the original datasets. | United Nations M49 standard geoscheme: https://unstats.un.org/unsd/methodology/m49 | NA | NA |
| **crop.vernacularName** | Crop, as listed by the border inspector | The border inspector listed crop either by vernacular or scientific name. 427 crops, or multiple crops, were listed by inspectors | NA | Either the vernacular or scientific name was added, as needed. | Spelling mistakes were corrected and names were edited for consistency (e.g. grapes instead of grapevine, red grapes, white grapes, table grapes, etc.) |
| **crop.scientificName** |  |  |  |  |  |
| **crop.family** | Taxonomic grouping | This was not part of the original datasets. | GBIF ^1^ (Accessed 28 Aug 2020) | Where multiple crops were listed, order and/or family was included if these were the same and recorded as "multiple" if they were not. | NA |
| **crop.order** |  |  |  |  |  |
| **crop.commodity** | Commodity type, as recorded by the border inspector | The border inspector choose commodity from a list of 9 categories, including the option to record it as "other" with or without further explanation. | "List of import commodity types", Table 3 | The original 9 categories were expanded to 30, and assigned to category based on what was recorded as commodity and crop as well as any additional laboratory records or expert opinion (e.g. the original category "cuttings" was expanded to accommodate cut flowers, budwood, etc.) | NA |
| **importer.code** | A unique alphanumerical identifier for importer | When included, importer or exporter name with or without address. Importer and exporter information was listed for 90% and 34% of samples, respectively. | NA | An alphanumerical identifier was generated based on chronological appearance in the dataset. | Anonymization of this data is necessary to protect the confidentiality of the trading companies. |
| **exporter.code** | A unique alphanumerical identifier for exporter |  |  |  |  |
| **Contaminant.Insecta  (12 columns)** | Contaminant insects arranged by order, plus one column for unidentified Insecta. | Identity of contaminant arthropods was recorded, sometimes along with notes on taxonomy, biology and quarantine status. Information on insects, mites and other arthropods were sometimes recorded together and sometimes apart (depending on original dataset). From ca. 2005, all arthropods were recorded, regardless of quarantine status. | GBIF ^1^ (Accessed 29 July 2020); recorded in Table 2. | Where taxonomy could not be resolved using GBIF, the taxonomy originally recorded was retained. | NA |
| **Contaminant.Acari  (11 columns)** | Contaminant Acari (mites & ticks) arranged by order, plus two suborders in Sarcoptiformes, four superfamilies in Trombidiformes, and one column for unidentified Acari. |  |  |  | A number of mite species were not listed on GBIF. |
| **Contaminant.**  **Arthropoda (not covered above)  (5 columns)** | Other contaminant arthropods arranged by phylum, class or order, depending on level of identification available. |  |  |  | NA |
| **contaminant.**  **Mollusca**  **(4 columns)** | Contaminant molluscs arranged by order, plus one column for unidentified Gastropoda. | Molluscs were recorded along with either insects or mites. | GBIF ^1^ (Accessed 29 July 2020); recorded in Table 2. |  | NA |
| **contaminant.**  **Nematoda (6 columns)** | Two classes, arranged according to order (5 columns) plus one column for unidentified Nematoda. | Only plant-feeding nematodes were routinely recorded, with saprophytic groups either recorded as a generic group (e.g. "saprophytic nematodes") or not at all. | GBIF ^1^ (Accessed 29 July 2020); recorded in Table 2. |  | NA |
| **contaminant.Fungi (23 columns)** | Three phyla and eight classes, arranged according to order (21 columns) plus three columns for unidentified Ascomycota, Basiodiomycota and Fungi. | Most fungi were identified to genus, with only possible quarantine specimens identified to species. Plant-pathogenic fungi were routinely recorded, with saprophytic fungi sometimes recorded. | GBIF ^1^ (Accessed 29 July 2020); recorded in Table 2. | Major taxonomic revision in mycology is currently underway to synchronise names between anamorph and teleomorph forms, thus classification may change in the future. |  |
| **contaminant.**  **Chromista (1 column)** | One class |  |  |  | Chromista is traditionally treated along with kingdom Fungi, and was previously considered the same kingdom. |
| **contaminant.Bacteria (9 columns)** | Three phyla and four classes, arranged according to order. | Only plant-pathogenic bacteria as detected by targeted testing were recorded. | GBIF ^1^ (Accessed 29 July 2020); recorded in Table 2. | Where taxonomy could not be resolved using GBIF, the taxonomy originally recorded was retained. |  |
| **contaminant.Virus (7 columns)** | Arranged according to family (6 columns), plus one column for unidentified viruses. | In most cases, only viruses detected by targeted testing were recorded. Occasionally, unidentified viruses were detected by biological indexing and recorded. | ICTV ^2^ |  |  |
| **contaminant.**  **Phytoplasmas (1 column)** | One column for phytoplasma tests | Tests for phytoplasmas were recorded, although none were detected. | NA | NA |  |

### References

1. GBIF.org. GBIF Home Page. https://www.gbif.org (2020).

2. Lefkowitz, E. J. *et al.* Virus taxonomy: the database of the International Committee on Taxonomy of Viruses (ICTV). *Nucleic Acids Res.* **46**, D708–D717 (2018).
